# Nanoparticles of zinc oxide defeat chlorpyrifos-induced immunotoxic effects and histopathological alterations

**DOI:** 10.14202/vetworld.2019.440-448

**Published:** 2019-03-22

**Authors:** Sara S. Essa, Eiman M. El-Saied, Osama S. El-Tawil, Inas M. Gamal, Sahar S. Abd El-Rahman

**Affiliations:** 1 Immune Section, Research Institute for Animal Reproduction, Cairo, Egypt; 2 Department of Toxicology, Forensic Medicine and Veterinary Regulations, Faculty of Veterinary Medicine, Cairo University, Egypt; 3 Department of Pathology, Faculty of Veterinary Medicine, Cairo University, Cairo, Egypt

**Keywords:** acetylcholinesterase, chlorpyrifos, immune system, pathology, zinc oxide nanoparticles

## Abstract

**Background and Aim:**

Chlorpyrifos (CPF) is a widely used organophosphate insecticide. Nanoparticles of zinc oxide (ZnO NPs) physically showed effective adsorbing property for some insecticides. The study was conducted to estimate the potential effect of ZnO NPs against CPF toxicity.

**Materials and Methods:**

Four groups of male rats were used; control group and three groups received drinking water contained 75 mg/L CPF, combined 75 mg/L CPF and 200 mg/L ZnO NPs, and 200 mg/L ZnO NPs, respectively.

**Results:**

CPF significantly decreased macrophage activity, serum lysozyme activity, and levels of interleukin-2 (IL-2) and IL-6; increased the percentage of DNA degeneration on comet assay of lymphocytes and significantly elevated hepatic and splenic malondialdehyde contents; and decreased their glutathione contents. The liver and spleen showed marked histological alterations after exposure to CPF with decreased expression of acetylcholinesterase. The coadministration of ZnO NPs ameliorated most of the undesirable effects of CPF, through elevation of macrophage and serum lysozyme activities, increased the levels of IL-2 and IL-6, corrected the oxidative stress markers, and alleviated most of the adverse effect exerted by CPF in liver and spleen tissues.

**Conclusion:**

The addition of ZnO NPs to CPF-contaminated drinking water may be useful as a powerful antioxidant agent against toxic damage induced by CPF particularly in individuals who are on daily occupational exposure to low doses of CPF.

## Introduction

Commercial insecticides used in agricultural and non-agricultural purposes have the potential to cause significant human and animal illnesses through direct or indirect exposure during application. Due to the extensive use of insecticides, they persist in soil, surface waters, air, and agricultural products [1]. Chlorpyrifos (CPF) is a well-known organophosphorothioate insecticide that is used for agricultural and non-agricultural areas. It is a broad-spectrum insecticide used to kill a wide range of insects [2,3]. CPF inhibits acetylcholinesterase (ACHE) enzyme of the nervous system necessary for proper function of the nervous system. Symptoms associated with the CPF poisoning including; nausea, vomiting, diarrhea, headache, convulsions, coma, and death in severe conditions. Long period exposure to CPF results in serious harm effects to the nervous system, respiratory tract, and cardiovascular systems. CPF metabolites persist in the environment for a long period; therefore, it becomes a public concern. CPF can be oxidized by various oxidizing agents, which give CPF-oxon after the replacement of sulfur by oxygen in the thiophosphoryl bond [4]. CPF-oxon is more toxic compared to its parent compound [5].

Nanotechnology offers fast and effective solu- tions for environmental cleanup. It has attracted considerable interest of both scientific and industrial communities because it is often described as an emerging technology capable of revolutionizing approaches to common problems [6]. Nanostructured membranes with size-selective pores may provide efficient ways of separating solutes from water [7]. Besides filtration, which is generally energy intensive, the removal of contaminants by sequestration (adsorptive remediation) or degradation to less toxic products (reactive remediation) may represent an effective alternative. Nanomaterials possess a very large surface-to-volume ratio that favors interaction with their environment. For example, nanomaterials have the potential to effectively adsorb molecules or catalyze chemical reactions at their interface [8]. Shahram *et al*. [9] showed that nanoparticle (NPs) form of zinc oxide (ZnO) was effective adsorbing agent for permethrin insecticide in water system, and the amount of reduction is related to permethrin concentration. For this reason, ZnO in the NP form could be an eminent candidate for preventing CPF adverse effects.

As per our knowledge, no work has been reported concerning the role of ZnO, in the NP form against CPF toxicity in *in vivo* system. Hence, this work aimed to investigate the potential effects of this formulation to relieve the toxic effect of CPF on immune system prospecting its application industrially and medically to remove this insecticide from animal’s water to decrease its toxicity. In addition, this work was employed to confirm that ZnO NPs have no harmful effects when it is added to the drinking water of the animals.

## Materials and Methods

### Ethical approval

The Institutional Animal Care and Use Committee (IACUC) of Cairo University approved the design of the experiment (IACUC protocol number: CU-II-S-50-17).

### Chemicals

CPF was provided from Central Agricultural Pesticide Laboratory, National Center for Agricultural Research, Ministry of Agriculture, Giza, Egypt.

ZnO NPs (100 nm) were purchased from Nano-Tech, Dreamland, 6^th^ October, Giza, Egypt. Other chemicals were purchased from Sigma-Aldrich Chemicals Co., St. Louis, MO, USA, and Cusabio Biotech Co. Ltd.

### Animals

Sixty mature male Sprague Dawley rats weighing (180±10 g) were used in this investigation. They were obtained from an animal house belonging to the Department of Veterinary Hygiene and Management, Faculty of Veterinary Medicine, Cairo University. Animals were maintained in plastic cages, fed on standard commercial pelleted feed and water that supplied *ad libitum*. They were observed for health status and were acclimated to the laboratory environment for 2 weeks before use.

### Experimental protocol

Rats were randomly divided into four equal groups. The first group served as control, the second group received drinking water contained 75 mg/L CPF, the third group received drinking water contained 75 mg/L CPF and 200 mg/L ZnO NPs, while the fourth group received drinking water contained 200 mg/L ZnO NPs. The experiment was extended for 9 weeks. The selected concentration of CPF was nearly equivalent to^1^/_20_ of the LD_50_ [10], while the selected concentration of ZnO NPs was nearly equivalent to 30 mg/kg body weight which considered of no observable adverse effects [11]. Blood samples were collected every 3 weeks as will be mentioned, while at the end of the experimental period, animals of all groups were sacrificed under anesthesia using ketamine and xylazine (40 mg/kg and 5 mg/kg, respectively) intraperitoneally, then liver and spleen were carefully dissected out, blotted free of blood and each organ was divided into two parts, one part was kept in 10% buffered neutral formalin for histopathological examination and the other was kept at −20°C for further assessment of antioxidant markers.

### Immunological assessment

Every 3 weeks, ten animals from each group were randomly selected for blood sample collection from the retro-orbital venous plexus under anesthesia using the mix of ketamine and xylazine (40 mg/kg and 5 mg/kg, respectively) IP. Blood samples were used for the assessment of macrophage activity after 1 and 2 h and comet assay on lymphocytes. In addition, serum samples were used to estimate the activity of serum lysozyme and the titer of interleukin-2 (IL-2) and IL-2.

### Determination of macrophage activity

The amount of nitrite produced from isolated and cultivated macrophages during exposure to infection was determined by mixing the supernatant with the colorless Griess reagent forming a purple complex. The degree of the developed color was measured spectrophotometrically after 1 and 2 h using ELISA reader at 570 nm [12].

### Comet assay of lymphocytes

DNA single-strand breaks (frank strand breaks and incomplete excision repair sites) were investigated at the single cell level using single-cell gel electrophoresis as previously mentioned by Tice *et al*. [13].

### Determination of serum lysozyme activity

Lysozyme is an enzyme that is known to kill the organism through hydrolysis of their cell walls. Lysozyme enzyme was allowed to diffuse through the agarose gel containing a suspension of *Micrococcus lysodeikticus* as the substrate producing a clear zone ring of lysis on the agarose gel. At the end of the incubation period, the diameters of the clear zone rings were measured to the nearest 0.1 mm with an enlarger viewer (Kalesttad Laboratories., Inc., and Austin, TX). For each lysoplate, the lysozyme concentration in the samples was determined from a plotted standard curve against the corresponding clear zone ring diameter on the linear axis [14].

### Determination of serum IL-2 and IL-6

The quantitative sandwich enzyme immunoassay technique was employed for the determination of IL-2 according to Goldsmith and Greene [15] and IL-6 according to Hirano [16]. Antibodies specific for IL-2 or IL-6 have been precoated onto microplates. Standards and samples were pipetted into the wells and, if the samples contain any IL-2 or IL-6, it will be bound by the immobilized antibody. Any unbound substance was removed, and then, a biotin-conjugated horseradish peroxidase was added to the wells. Any unbound avidin-enzyme reagent was washed followed by the addition of a substrate solution to the wells. The developed color will be in proportion to the amount of IL-2 or IL-6 bound in the initial step. The color development was stopped, and the intensity of the color was measured.

### Determination of the antioxidant markers in liver and spleen tissues’ homogenates

Briefly, homogenization of liver and spleen tissue specimens was performed in 5-10 ml cold buffer (50 mM) of potassium phosphate (pH 7.5) and 1 mM EDTA or 1 mM EDTA per gram tissue for the determination of the lipid peroxidation marker; malondialdehyde (MDA) and glutathione (GSH) levels, respectively. Following centrifugation of the obtained homogenates at 5000 rpm for 20 min at 4°C, the supernatants were aspirated and transferred into Eppendorf Tubes and then preserved at −80°C until used. Both MDA and GSH contents were assessed in the hepatic and splenic tissue homogenates according to Ellman [17] and Ruiz-Larrea *et al*. [18], respectively.

### Histopathological and immunohistochemical evaluation

After fixation of liver and spleen specimens in 10% buffered neutral formalin for 72 h, the specimens were washed, dehydrated in graded series of alcohol and cleared in xylol, and then embedded in paraffin. Serial sections of 4-5-µm thickness were obtained and stained with hematoxylin and eosin [19]. Electric light microscope Olympus BH2 (Tokyo, Japan) was used for the histopathological examination of hematoxylin and eosin sections.

Expression of ACHE in paraffin sections of hepatic tissue of all groups was demonstrated immunohistochemically according to the method described by Hsu *et al*. [20] using avidin-biotin-peroxidase (3,3-diaminobenzidine tetrahydrochloride [DAB], Sigma Chemical Co.).

After incubation of liver sections with monoclonal antibody for ACHE (Dako Corp, Carpinteria, CA) and reagents of avidin-biotin-peroxidase method (Vectastain ABC Peroxidase Kit, Vector Laboratories), antigen-antibody complex was detected. The immunoreactive cells were visualized using chromagen DAB (Sigma Chemical Co.). Following examination, the intensity of immunohistochemical staining of ACHE was quantified in random five high microscopic fields as an optical density using image analysis software (Image J, 1.46a, NIH, USA).

### Statistical analysis

The results were expressed as mean±standard error of the mean. Data were analyzed using one-way analysis of variance followed by Duncan’s multiple range test and GraphPad software CoStat. p<0.05 was considered statistically significant.

## Results

### Macrophage phagocytic activity

**Figure-1:**
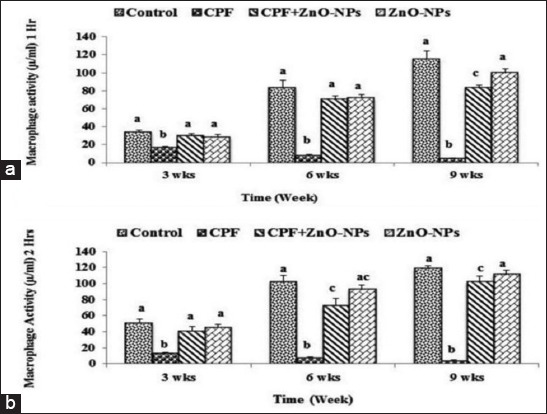
Mean values±standard error of the macrophage activity (µM/ml) after 1 h (a) and 2 h (b) from its cultivation of control and experimental rats which received water contained 75 mg/L chlorpyrifos and/or 200 mg/L zinc oxide nanoparticles. Columns have different scripts at the same time are significantly different at p≤0.05 (n=10).

Macrophages’ activity of rats which received water contained 75 mg/L CPF after 1 and 2 h from its cultivation showed a significant (p<0.05) decrease compared to the control group along the three periods (after 3, 6, and 9 weeks) of assessments, while macrophages’ activity of those rats which received water contained CPF and 200 mg/L ZnO NPs was significantly higher than that of CPF-intoxicated group along the experimental period. The activity of macrophages of ZnO NP-administrated group showed non-significant differences compared with that of control sets in the three periods’ assessments (Figure-1a and b).

### Comet assay of lymphocytes

**Figure-2:**
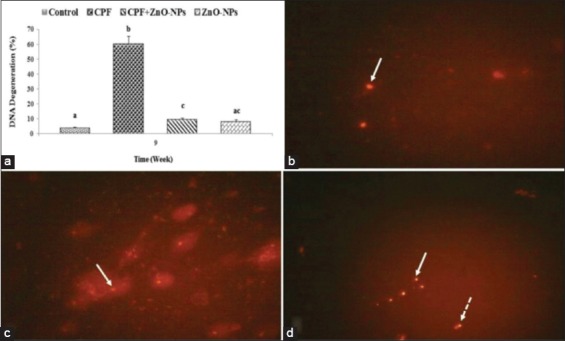
Comet assay of lymphocytes. (a) The mean values±standard error of the of DNA degeneration percentage of lymphocyte in all groups. (b-d) Nuclei of the lymphocytes of; (b) control rat have no tail (arrow) revealing intact DNA, (c) rats which received water contained chlorpyrifos (CPF) have head and tail (arrow) revealing many DNA breaks, (d) rat which received water contained combined CPF zinc oxide nanoparticles revealed many intact DNA that has no tail (arrow) and few DNA breaks which have head and tail (dashed arrow). Columns have different scripts at the same time are significantly different at p≤0.05 (n=10).

DNA degeneration percentage in control and the other treated groups is presented in Figure-2a. The n uclei of the lymphocyte cells of the control group in the last week of the experiment (9^th^ week) had no tail revealing intact DNA (Figure-2b). While those of rats received CPF, their DNA showed significant (p<0.05) high degeneration percentage compared with the control group and many nuclei appeared with head and tail (Figure-2c). On the other hand, rats which drank water contained CPF and ZnO NPs (Figure-2d) revealed significant (p<0.05) lower DNA degeneration percentage compared with that observed in the CPF-intoxicated group. ZnO NP-administrated group revealed a non-significant difference from the control group.

### Serum lysozyme activity

**Figure-3:**
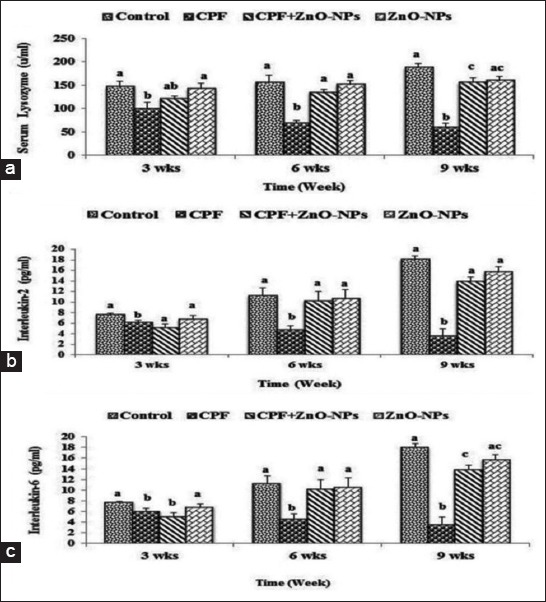
Mean values±standard error of (a) serum lysozyme activity (u/ml), (b) interleukin-2 (IL-2) titer (pg/ml), and (c) IL-2 (pg/ml) in control and experimental groups which received water contained 75 mg/L chlorpyrifos and/or 200 mg/L zinc oxide nanoparticles. Columns having different scripts at the same time are significantly different at p≤0.05 (n=10).

Serum lysozyme activity of CPF-intoxicated group showed significant (p<0.05) decrease when it compared with that of controls at each time point. The administration of ZnO NPs to CPF-intoxicated rats significantly (p<0.05) increased the lysozyme activity compared with that of CPF-intoxicated group particularly at the 6^th^ and 9^th^ weeks of assessment. Lysozyme activity of rats which received water contained ZnO NPs showed no difference from that of the controls (Figure-3a).

### Serum IL-2

A gradual increase in serum IL-2 titer was recorded in the serum of control group along the periods of assessment (3^rd^, 6^th^, and 9^th^ weeks). However, serum IL-2 titer of rats intoxicated with CPF (75 mg/L) showed significant (p<0.05) decrease compared with that of control and the other experimental groups. Rats which received water contained combination of CPF and ZnO NPs showed a significant increase in their IL-2 titer compared to that of CPF intoxicated rats, while serum titer of IL-2 of rats which administrated ZnO NPs in their drinking water showed no difference from the control group along the experimental period (Figure-3b).

### Serum IL-6

Serum IL-6 titer of rats which administrated CPF showed a significant (p<0.05) decrease compared to that of controls at each time point. The titer of the group intoxicated with CPF and treated with ZnO NPs showed a significant (p<0.05) increase compared with that of CPF-intoxicated group except at the 3^rd^ week of the experiment. Serum IL-6 titer for ZnO NPs-treated group showed a non-significant difference from that of the control group along the whole period of the experiment (Figure-3c).

### Oxidative stress markers

**Figure-4:**
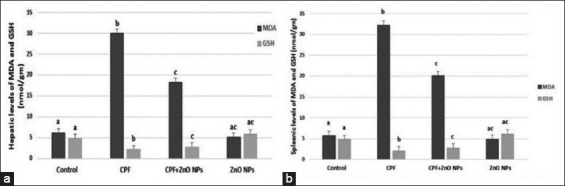
Mean values±standard error of hepatic and splenic contents of (a) malondialdehyde and (b) glutathione in control and experimental groups which received water contained 75 mg/L chlorpyrifos and/or 200 mg/L nanoparticles of zinc oxide. Columns having different scripts at the same time are significantly different at p≤0.05 (n=10).

Significant (p<0.05) elevation of both hepatic and splenic MDA levels (Figure-4a) accompanied with significant (p<0.05) reduction in their GSH content (Figure-4b) was observed in CPF-intoxicated rats compared with controls and those received ZnO NPs in their drinking water. The coadministration of ZnO NPs with CPF in the drinking water of rats exhibited a significant (p<0.05) recuperation of that altered oxidative stress markers by significant (p<0.05) decrease in MDA level and a significant increase in GSH content.

### Histopathological and immunohistochemical findings

**Figure-5:**
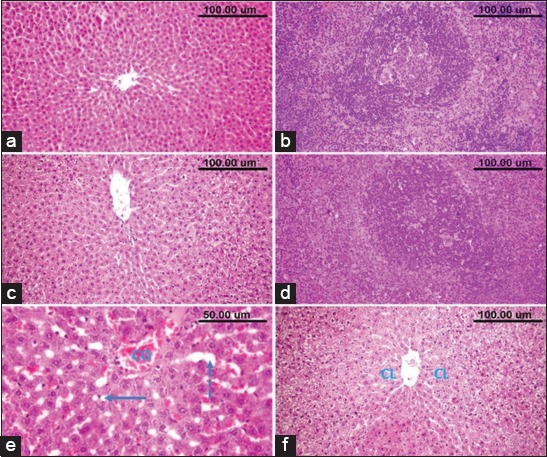
The liver and spleen of normal control (a and b) and nanoparticles of zinc oxide-administrated rats (c and d). (e and f) The liver of chlorpyrifos-administrated rats showing; (e) congestion of central vein (CO), dilatation of hepatic sinusoids (dashed arrow) and (e) and (f) centrilobular (CL) hepatocellular degeneration and necrosis.

**Figure-6:**
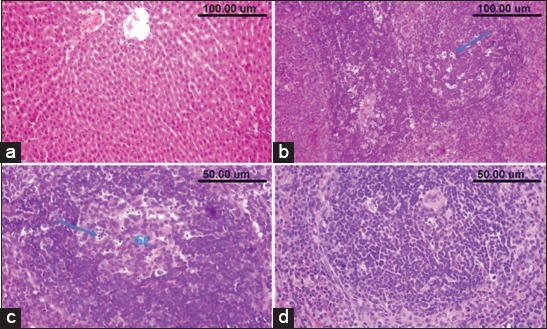
(a) The liver of chlorpyrifos (CPF) and nanoparticles of zinc oxide (ZnO NPs) administrated rat showing minimal degenerative changes in the hepatic parenchymal cells. (b and c) Spleen of rat which received CPF showing lymphocytic depletion with many tingible body macrophages (arrow) and lymphocytes apoptosis in the follicles, loss of the lymphocytes in the germinal center with appearance of the underlying reticular mash. (d) The spleen of combined CPF and ZnO NP-administrated rat showing minimal lymphocytic necrosis.

Microscopic examination of the liver and spleen of normal control (Figure-5a and b) rats and those administrated ZnO NPs revealed normal histological structure (Figure-5c and d). Liver sections of CPF-administrated rats revealed congestion of the central veins and sinusoids (Figure-5e). Hepatic sinusoids were dilated and showed leukocytosis. Widespread hepatocellular vacuolar degeneration and necrosis were evident particularly in the centrilobular area (Figure-5f). The necrotic cells appeared with pyknotic or karyorrhectic nuclei or without any nuclear structure. Livers of rats received water contained combined CPF and ZnO NPs showed good protection of the hepatic parenchymal cells against the harmful effect of CPF with the only appearance of minimal degenerative changes (Figure-6a). The spleen of rats which received CPF in their drinking water showed a marked lymphocytic depletion of the white pulp lymphoid follicles with increased number of the tingible body macrophages and lymphocyte apoptosis (Figure-6b). Marked loss of lymphocytes in the germinal center with the appearance of the underlying reticular mash was observed (Figure-6c). The central arteriole showed thickened wall and focal areas of hyalinization. However, the spleens of combined CPF and ZnO NPs administrated rats showed minimal lymphocytic degeneration and necrosis (Figure-6d).

**Figure-7:**
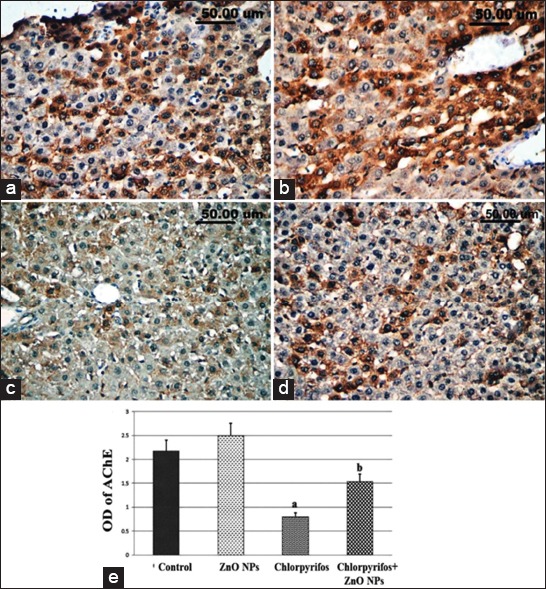
Acetylcholinesterase (ACHE) immune expression in the liver of control (a) and experimental groups which received water contained 200 mg/L nanoparticles of zinc oxide (ZnO NPs) (b), 75 mg/L chlorpyrifos (c), and combined CPE and ZnO NPs (d). (e) The quantitative image analysis of ACHE immune expression in various groups expressed as optical density in 5 microscopic fields. a: significantly different from control group at p<0.05, b: significantly different from chlorpyrifos group at p<0.05.

ACHE showed marked immunopositivity in the livers of control (Figure-7a) as well as ZnO NP (Figure-7b) administrated rats, while the livers of rats which received CPF in their drinking water showed a significant (p<0.05) decreased expression of hepatic ACHE (Figure-7c). The latter decrease was significantly (p<0.05) restored on the combined use of ZnO NPs with CPF (Figure-7d) as detected by the quantitative analysis of ACHE immune expression in various groups (Figure-7e).

## Discussion

CPF is a broad-spectrum anticholinesterase insecticide, utilized extensively in agriculture and residential pest control throughout the world [21]. Monitoring studies conducted in different countries revealed the presence of residues of this insecticide in different varieties of food commodities [22,23]. Moreover, CPF was also cytotoxic and immunotoxic even at lower concentration [24].

ZnO NPs physically showed effective adsorbing property for some insecticides [9]. The present study was conducted to follow up the protective efficacy of ZnO NPs (100 nm) against CPF immunotoxicity in male rats when they left to gain access to CPF-contaminated drinking water.

In the current study, cellular immune response of male rats was significantly reduced as a result of drinking water contaminated with 75 mg/L CPF. The recorded immunotoxic effects of CPF could be attributed to its direct toxic effect on blood cells as recorded by significant low percentages of phagocytic activity of macrophages after 1 and 2 h from its cultivation and serum lysozyme activity or indirectly on the hematopoietic organs as observed by the histopathological and immunohistochemical alterations in liver and spleen.

Despite CPF is ACHE inhibitor, its adverse effects are not only confined to cholinergic system but also to other body systems. Many studies elucidated that CPF exerted several adverse effects including hemotoxicity, immunological abnormalities as well as hepatic and renal dysfunctions [25-27]. Other studies revealed leukopenia apparently due to lymphopenia, neutropenia, and monocytopenia in the CPF-administrated animals [25,28] and oxidative stress as well [29]. In the present investigation, immunotoxic manifestations induced by CPF may be associated with the enhanced production of reactive oxygen species (ROS), which cause damage to various membrane components of the cells of the immune system. Mansour *et al*. [27] identified ROS as a cause of toxic effects exerted by organophosphorus pesticides. These ROS are responsible for inducing oxidative stress in the tissues and chronic permanent damage.

Comet assay of the lymphocytes in the present study showed a general trend of increased DNA degeneration in the CPF-intoxicated rats when it compared with the control and the other experimental groups. ROS produced due to CPF toxicity [21] could be elicited detrimental DNA damage. Many reports have identified two potential cellular targets for CPF, cell signaling cascades from one side and the expression and function of gene transcription factors from the other side [30]. Oxidative stress is known to be a key factor in several diseases. In fact, one of the molecular mechanisms of some pesticides’ toxicity seems to be lipid peroxidation; as a consequence, these compounds can disturb the biochemical and physiological functions of the immune system cells as well as the liver and kidney [31]. The current results illustrated that CPF had a strong inhibitory effect on both IL-2 and IL-6; these cytokines stimulate T-lymphocyte proliferation and stimulate the activities of T-helper cells and cytotoxic T-cells. They also induce the differentiation of Th1 cells and inhibit the differentiation of Th2 cells to regulate immune response [32]. Similar studies showed that CPF had an inhibitory effect on both IL-2 and interferon-γ production on rats [28] and also CPF suppressed T-lymphocyte proliferation and reduced serum IgG and IgM levels in rats [26,33].

As demonstrated by the microscopic examination, CPF exerted marked histological alterations in the examined liver and spleen tissues which could be attributed to the formation of ROS by CPE exposure. It had been reported that biologically active substances such as pesticides boost the formation of ROS which is responsible for the actuating oxidative stress in the tissues and subsequently their damage [34,35]. In addition, some studies identified ROS as a cause of toxic effects exerted by CPF as it increased oxidative stress in different tissues and organs [36,37] which supported with our results of increased hepatic and splenic contents of MDA and decreased their GSH contents. However, the addition of ZnO NPs to the drinking water contained CPF and markedly relieved the tissue alterations exerted by CPF. In this regard, several studies have demonstrated that zinc possesses antioxidant properties and consequently can protect the body cells from oxidative damage induced by certain xenobiotics [38]. In addition, zinc plays an essential role in cellular GSH regulation which is a vital process to cellular antioxidant defense, thus protecting the cell against ROS induced by OP pesticides [31]. The protective effect of zinc could be achieved through its interaction with cell membranes, thus stabilizing them against various damaging effects, including those caused by oxidative injuries.

ACHE expression was significantly decreased in the liver of CPF-administrated rats. CHE is known to be synthesized mainly in hepatocytes and then secreted into the bloodstream [39]. As a result of liver dysfunction, CHE synthesis and activity will be declined in comparison with the other serum hepatic function enzymes that related to the clinical assessment of liver function whose levels increased due to their increased release from their cellular sources following damage of cell membrane [40].

Like other organophosphorus insecticides, CPF is an ACHE enzyme inhibitor which leads to the accumulation of acetylcholine and results in excessive stimulation of postsynaptic receptors and consequent signs of toxicity [41]. CPF does not directly inhibit ACHE enzyme; it is first metabolized to the corresponding oxygen analog (CPF-oxon), a more potent inhibitor of ACHE enzyme. The activation of CPF into CPF-oxon is mediated by cytochrome P_450_ mixed function oxidases, primarily within the liver [42]; however, extrahepatic metabolism has been reported in other tissues including the brain [21]. Short-term exposure of CPF in rats caused a significant inhibition of ACHE enzyme activity in different tissues including the liver, kidney, and spleen [27].

The current results demonstrated that all the recorded CPF adverse effects on the immune system of male rats were significantly relieved by addition of ZnO NPs (200 mg/L) to the contaminated water. These findings suggested that ZnO NPs directly alleviated these effects by adsorbing CPF and consequently preventing it from inducing its toxic effect on the immune system. That adsorbing process is chiefly occurred as a result of the electrostatic attraction between negatively charged CPF anions and positively charged surface of the sorbent [43]. Another mechanism of ZnO NPs for alleviating the toxic effects of CPF in this research could be attributed to the antioxidant effect of ZnO NPs. As one of the essential nutrients, zinc can protect against oxidative damage caused by certain xenobiotics and thus may have antioxidant properties. It acts as a cofactor for important enzymes involved in the proper functioning of the antioxidant defense system [38,44]. Exposure to ZnO-NPs in this study does not affect the exploratory behaviors of male rats. A similar finding was recorded by Soheili *et al*. [45] and Amara *et al*. [46].

## Conclusion

The present results concluded that the coadministration of ZnO in the NP form to CPF-contaminated drinking water restored the viability and function of the immune cells, the oxidative stress markers, the histopathological alterations as well as the immune-expression of ACHE. These results give light on the beneficial use of ZnO NPs in CPF-contaminated water and in individuals who are occupationally on daily exposure to low concentration of such insecticide.

## Authors’ Contributions

EME and OSE designed the study and analyzed the data. IMG and SSAE contributed to the reagents/materials/analysis tools and analyzed the data. SSE contributed to the reagents/materials/analysis tools, collected the material, and analyzed the data. All authors read and approved the final manuscript.
